# Psychosocial stress-associated alterations in microRNAs and their relevance for metabolic diseases: A systematic review

**DOI:** 10.1016/j.cpnec.2026.100364

**Published:** 2026-07-17

**Authors:** Nele Reinsberg, Ankush Borlepawar, Parvana Hajieva, Anett Müller-Alcazar

**Affiliations:** a Department of Human Medicine, Medical Faculty, MSH Medical School Hamburg - University of Applied Sciences and Medical University, Am Kaiserkai 1, Hamburg, 20457, Germany; b ICAN Institute for Cognitive and Affective Neuroscience, MSH Medical School Hamburg - University of Applied Sciences and Medical University, Am Kaiserkai 1, Hamburg, 20457, Germany; c Cellular Adaptation and Bioenergetics Group, Institute for Translational Medicine (ITM), MSH Medical School Hamburg - University of Applied Sciences and Medical University, Am Kaiserkai 1, Hamburg, 20457, Germany; d Department of Psychology, Faculty of Human Sciences, MSH Medical School Hamburg - University of Applied Sciences and Medical University, Am Kaiserkai 1, Hamburg, 20457, Germany

**Keywords:** Psychosocial stress, microRNA, Gene expression, Epigenetics, Metabolism, Metabolic diseases, Systematic review

## Abstract

**Background:**

Psychosocial stress is associated with an increased risk of physical and mental illness. The diverse molecular mechanisms involved in this process are still not fully understood. MicroRNAs (miRNAs) may be involved not only in stress reactivity but also in the development of diseases. This systematic review aims to summarize psychosocial stress-related changes in miRNA expression in humans. Implications for metabolic diseases are investigated in an exploratory manner.

**Methods:**

The review was pre-registered at PROSPERO (CRD42024590242). A systematic literature search was conducted in four databases (EBSCO, PSYNDEX, PubMed, Web of Science), to find original peer-reviewed studies that investigate miRNA changes in response to psychosocial stress in adults. Quality was assessed for all included studies.

**Results:**

Overall, 12 studies with N = 489 participants were included. The age of participants ranged from 18 to 84 years. Various methodological approaches were used to elicit and assess psychosocial stress, such as laboratory and academic stressors or self-reports. miRNAs were collected from blood, breast milk, and saliva over different time periods. 34 miRNAs were identified as being differentially expressed in response to psychosocial stress.

**Conclusion:**

Several miRNAs were altered in response to psychosocial stress in humans. However, most of the identified miRNAs were detected in only one study, and the substantial heterogeneity in study designs poses a challenge for direct comparisons of the results. The investigation of stress-related miRNA changes is promising for future prevention and treatment of diseases and could contribute to a better understanding of miRNA-regulating mechanisms in metabolic diseases.

## Highlights

• We included 12 studies on miRNA changes in response to psychosocial stress.
• miRNAs have been assessed in blood, breast milk, and saliva.
• Stress was measured using laboratory/academic stressors, self-reports, and biomarkers.
• We identified 34 altered miRNAs in response to stress during various time periods.
• More research is needed to develop non-invasive stress-associated miRNA biomarkers.

## 1 Introduction

Stress arises if an individual's physiological or psychological integrity is perceived as threatened, eliciting physiological and/or behavioral reaction [1]. Physical stressors (e.g., prolonged exercise, electric shock) and psychological stressors (e.g., public speaking) [2], their interpretation, as well as stress responses vary across individuals [1], especially in the case of psychological stress due to a lack of control or predictability [3].

For maintenance of homeostasis, the activation of stress response induces endocrine alterations. The central effectors of the stress response are a rapid activation of the sympatho-adrenomedullary system (SAM), followed by a delayed activation of the hypothalamic-pituitary-adrenal-axis (HPA axis). During acute stress, Corticotropin-releasing hormone (CRH) is synthesized in the hypothalamus and released into the hypophysial portal system, inducing the release of adrenocorticotropic hormone (ACTH) in the adenohypophysis. Once ACTH is released into the bloodstream, it stimulates cortisol synthesis and secretion in the adrenal cortex, which can further inhibit the activity of the HPA axis via a negative feedback loop [4,5].

While stress per se is not always harmful, an adaptive response to stress can be beneficial, such as preparing for fight or flight reaction. Especially chronic stress is associated with various psychological and physiological illnesses such as depression [6], cardiovascular disease [7], or metabolic diseases [6]. However, the underlying molecular mechanisms are still largely unknown. This is particularly concerning given that stress has been highly prevalent during the recent COVID-19 pandemic [8] and that psychosocial stress, to which individuals are increasingly exposed in today's society, tends to last longer and cannot be relieved as quickly as stress due to acute physical threats [9].

Psychosocial stress has been extensively investigated across various disciplines in recent years, resulting in multiple concepts [10]. Studies investigating psychological stress typically concentrate either (1) on environmental events that are challenging one's coping skills, or (2) on individuals' reactions (e.g., perceived stress or negative effects) to events suggesting this overload, [11]. Psychosocial stress can arise from interpersonal situations that involve social threat, such as racism and ethnic discrimination [12] or social evaluation. Among the diverse methods used to assess psychosocial stress, one of the most established procedures for experimentally inducing acute psychosocial stress is the Trier Social Stress Test (TSST), which combines uncontrollability and social-evaluation threat [13,14].

Not only stress, but overweight and obesity are ubiquitous, with prevalences continuously increasing, affecting 37.5% of the global population [15]. Being overweight or obese can increase the risk for mortality and is associated with numerous co-morbidities like type 2 diabetes mellitus (T2DM), cardiovascular diseases, or different types of cancer [16]. Moreover, obesity accounts for high economic burden due to elevated healthcare costs [17]. In this context, stress may play a pivotal role in the development of obesity through multiple behavioral, physiological or biochemical mechanisms, such as disrupted sleep and activity patterns or enhanced fat deposition driven by cortisol secretion [18]. Alterations in the activity of the HPA axis may further mediate the relationship between stress and obesity [19].

Further, epigenetic modifications can contribute to metabolic processes, resulting in metabolic dysregulation as well, by various small molecules including microRNAs (miRNA, miR) [[20], [21], [22]]. Since their discovery in *Caenorhabditis elegans* in 1993 [23,24], the study of miRNAs has increasingly become the subject of scientific research. miRNAs are defined as small non-coding RNAs that can enhance or repress genes and thus regulate the expression and translation of target messenger RNA (mRNA) [25]. With more than 2000 miRNAs estimated in humans [26], miRNAs are assumed to target over 60% of human protein-coding genes [27]. miRNAs are being recognized as potent gene regulators, but with the full extent of their regulatory effects remaining incompletely understood, further research is required [28]. miRNA seems to play a key role in several biological functions as well as metabolic pathways like glucose and cholesterol metabolism or pancreatic islet function [29]. Accordingly, various miRNAs have been linked to a variety of different pathologies [30,31] and more specifically to several metabolic diseases and obesity [32,33]. Stress might influence the relationship between miRNAs and metabolic diseases by contributing to epigenetic modifications involved in metabolic regulation and obesity [34]. The involvement of miRNAs in the regulation of stress response pathways is attributed to the fact that they e.g., act (1) directly as an mediation element (2), as a signal modulator, (3) or as part of a negative/positive feedback loop [35]. Initial studies have already examined stress-induced alterations in miRNA expression [36,37] and their association with stress-related disorders, such as psychiatric conditions [38]. A standardized and robust detection method for miRNA is essential for future research efforts with miRNAs being detected in several body fluids like serum, plasma, urine, saliva [39] and even in breast milk or tears, wherein the amount and specificity of miRNAs can vary depending on the fluid type [40]. Together with their vast stability, the relative ease of extraction and detection and their reproducible nature coupled with their presence in non-invasively extractable body fluids, miRNAs present a promising non-invasive biomarker for multiple disease detection, prognosis and treatment [41]. In addition, many miRNAs are observed not only in single diseases but in many pathologies, and specific miRNAs are discussed as general disease biomarkers for cancer and non-cancerous diseases. Further, even miRNA-targeted therapeutics, like miRNA mimics (to elevate beneficial miRNA expression) or inhibitors (to inhibit miRNA activity linked to disease progression) are discussed [35,42] in diseases such as cardiovascular [42] and metabolic diseases [43]. With clinical trials investigating miRNA therapeutics, miRNA-based medication holds a promise, particularly for diseases currently lacking effective treatment options [44].

Moreover, specific combinations of miRNAs, referred to as miRNA signatures, may serve as more robust biomarkers; however, validation remains essential [30], as factors such as fluid type, and biological variations can influence miRNA expression [31].

While previous reviews have focused on the influence of miRNAs on metabolism, metabolic diseases or obesity e.g., Refs. [22,33,45] as well as the effect of miRNAs on stress pathways and stress-associated changes related to disease e.g., Refs. [35,37,38], this is, to our knowledge, the first systematic review focusing specifically on differentiated miRNA expression associated with psychosocial stress and its putative effect on metabolic disease in humans. Although an earlier review conducted in this area focused on altered miRNA expression in response to psychosocial stress [36], this systematic review aims to provide an up-to-date and systematic approach, focusing on metabolic diseases in an exploratory manner. Metabolic diseases were prioritized for the exploratory search due to the observed pathophysiological links between stress and conditions such as obesity [18,19], metabolic syndrome [46], T1DM and T2DM [47]. These associations are partially explained by shared underlying mechanisms, including HPA axis dysregulation [19], and insulin need/resistance [47]. This points to overlapping molecular mechanisms that may be reflected in overlapping miRNA expression profiles. The investigation of stress-related transcriptional changes holds significant promise for the prevention, detection, and treatment of various diseases. Exploring such associations could uncover novel biomarkers and therapeutic targets relevant to both stress-related disorders and metabolic conditions. In addition, validating saliva as a non-invasive source of RNA biomarkers has great potential to enhance the feasibility and accessibility of stress research.

## 2 Objectives

The aim of this review, according to the PICO framework, is to systematically investigate the impact of psychosocial stress (I) on altered miRNA expression levels (O) in adults (P). If applicable, pre- or no stress conditions or healthy norms will be compared (C). Further, we will emphasize stress associated miRNA alterations in metabolic diseases exploratory (O).

Accordingly, this systematic review addresses the following research questions:

1. Which miRNAs were differentially expressed in response to psychosocial stress in adults?
2. Exploratory: How do the differentially expressed miRNAs in response to psychosocial stress contribute to metabolism and metabolic diseases in adults?

## 3 Methods

In line with the principles of open science, this systematic review was pre-registered at PROSPERO (CRD42024590242) in September 2024. We made some minor and major amendments to the pre-registration, including shifting the focus away from metabolism to ensure a concise review. Further, we edited the review title and opted for two different quality assessment tools than originally described at PROSPERO, as they appeared to be better suited. This work was conducted according to the Preferred Reporting Items for Systematic Reviews and Meta-Analyses (PRISMA) guidelines [48].

### 3.1 Search strategy and study selection

A pilot search was conducted in August 2024 to identify our search strategy, which resulted in the search terms that all authors (NR, AB, PH, AMA) agreed on:

Title/abstract: (microRNA OR microRNAs OR miRNA OR miRNAs OR non coding RNA OR mir) AND (acute stress OR chronic stress OR psychosocial stress OR psychological stress OR psychologic stress OR social stress OR mental stress OR academic stress OR experimental stress OR nonchemical stress).

We considered four databases: EBSCO (APA PsycInfo, APA PsycArticles, MEDLINE complete, CINHAL complete, Psychology and Behavioral Sciences Collection), PSYNDEX, PubMed and Web of Science. Further, reference lists of all included eligible articles were searched for suitable studies by NR.

Our search in February 2025 resulted in a total of 528 articles. 108 duplicates were removed with 420 articles remaining for title and abstract screening. For inclusion, studies had to fulfil the following criteria: (1) written in English or German language, (2) original peer-reviewed study, (3) with quantitative methodology (4) and published before February 2025, (5) addressing miRNA expression in response to psychosocial stress (6) in humans (≥18 years). Animal (7) or plant studies (8) as well as in vitro studies (9) were excluded. Further, we excluded studies reporting solely on biological markers such as cortisol or inflammation markers without including psychological or experimental data on psychosocial stress (10). Additionally, studies investigating participants with posttraumatic stress disorder (PTSD) were excluded (11), as PTSD represents a clinically significant form of stress that does not necessarily result from psychosocial stress. Also, (12) studies exclusively examining psychosocial stress experienced in childhood (such as adverse childhood events (ACE) were excluded from our synthesis since our systematic review aimed to examine miRNA alterations associated with psychosocial stress exposure in adults only. ACEs can be conceptualized as potentially traumatic events, including abuse, neglect and household dysfunction, which can influence e.g., the neurological and endocrine systems, as well as epigenetic mechanisms, and possibly affecting long-term health [49]. Thus, ACEs are not directly comparable to psychosocial stressors experienced in adulthood, such as academic stress or acute laboratory stressors (TSST). For this review, psychosocial stress was defined as stress arising from social or interpersonal situations, commonly assessed through standardized experimental stress paradigms (e.g., TSST) or self-report questionnaires.

The screening of title and abstract was performed independently by AB and NR using Rayyan software [50]. Conflicts were resolved by discussion between AB and NR. Subsequently, 39 full texts were independently screened by AB and NR for eligibility. AMA and PH were consulted in case of conflicts, leading to the exclusion of 30 articles. The most common reason for exclusion was that no psychosocial stress was investigated, followed by wrong population (animal studies), in vitro studies and studies that did not investigate miRNA(s). One study reported group differences of stress-associated miRNAs in humans but did not provide information on specific individual altered miRNAs and was therefore excluded as well [51]. Another study was excluded since miRNA were analyzed from myometrial tissue which was collected during surgery for uterine fibroids [52]. Comparisons between miRNAs detected in body fluids and those expressed in tissue should be interpreted carefully. Despite a positive correlation and frequent communication observed between miRNAs in body fluids and human tissues [53], e.g., no coinciding serum and tissue miRNA patterns were found in patients with active Crohn's disease and active ulcerative colitis [54]. Also, the surgical procedure and underlying pathology may lead to procedure- and disease-related confounding, as dysregulated miRNA expression may contribute to the development of uterine fibroids [55]. Finally, the analysis of miRNA derived from tissue does not appear optimal for stress research, as tissue biomarkers are inherently invasive, whereas non-invasive biomarkers would be more suitable for this context.

Records identified from citation searching of included articles resulted in two eligible articles [56,57]. In September 2025, an updated, streamlined search was conducted. Since the systematic search had been conducted only seven months earlier, we decided to use the same search terms as in February 2025. NR searched PubMed for recently published articles after February 2025. This search yielded 28 articles, 21 of which were published after February 2025 and had not been included in our previous literature search. The titles and abstracts were screened by NR, resulting in the inclusion of one additional suitable article [58].

**Fig. 1:**
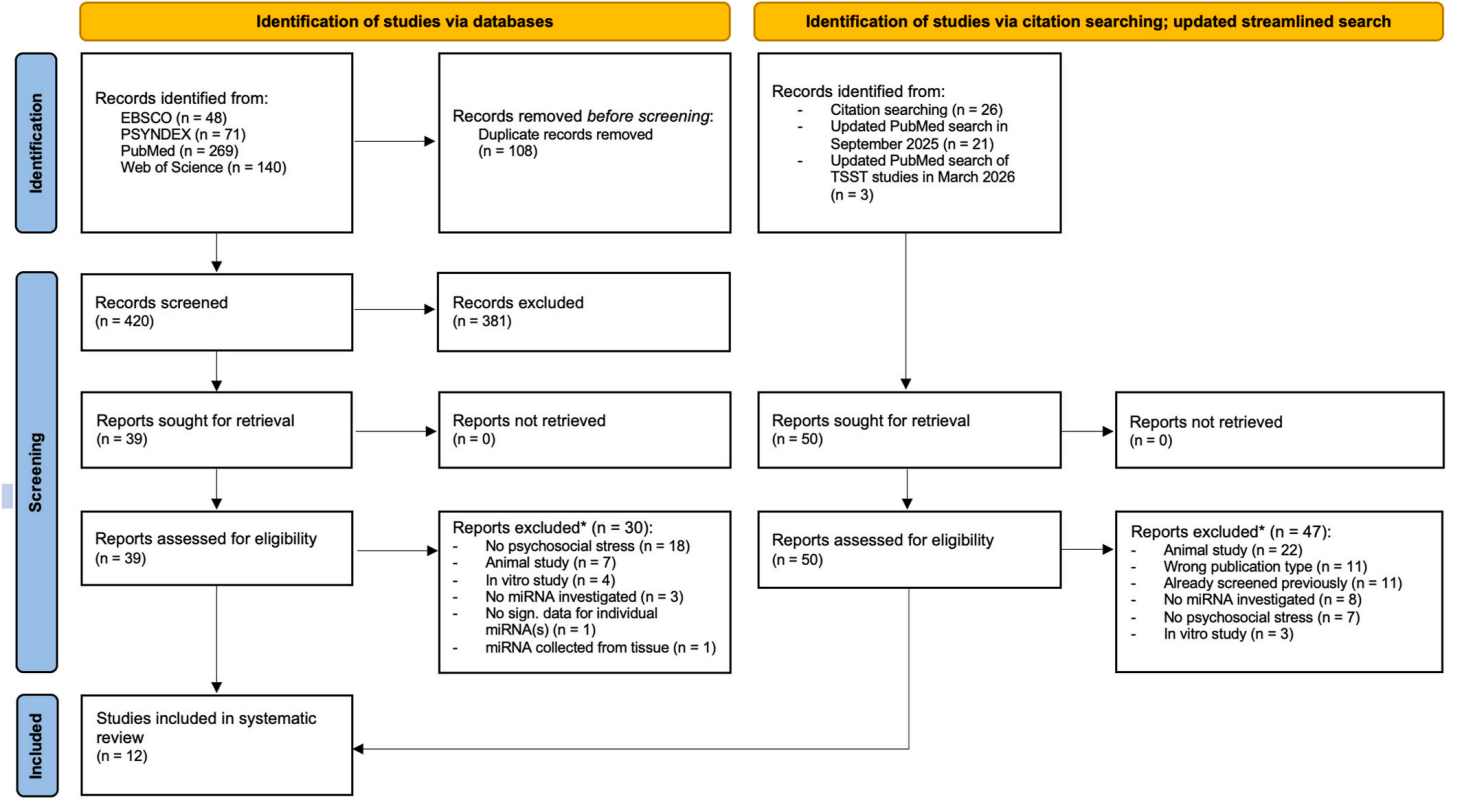
PRISMA 2020 flow diagram of the systematic literature search. Note. Source [48]; including searches of databases, reference lists of included studies and an updated streamlined search; *some studies met more than one exclusion criterion. Abbreviation: Sign. = significant.

As the TSST is considered the gold standard for inducing acute laboratory psychosocial stress, we conducted an updated, streamlined search in March 2026 in PubMed (Title/Abstract) using the following terms: (*Trier Social Stress Test OR TSST) and (microRNA OR microRNAs OR miRNA OR miRNAs OR non coding RNA OR mir)*. This search yielded three results, and no new eligible studies were identified. A total of 12 studies [[56], [57], [58], [59], [60], [61], [62], [63], [64], [65], [66], [67]] were included in this systematic review. The PRISMA flow diagram is presented in Fig. 1.

### 3.2 Data extraction

Data extraction was performed manually starting in March 2025 by one author (NR) using Microsoft Excel. Extracted information included general study data: (1) authors and year of publication, (2) title and journal, (3) DOI and (4) country of study realization. Extracted data on study design consisted of: (5) study type, (6) sample size, (7) participants' age, (8) gender ratio, (9) origin of participants, (10) participants' Body Mass Index (BMI), (11) participants' smoking status, (12) participants' health status, (13) medication intake, (14) menstrual cycle phase and intake of oral contraceptives, (15) participant's exercise level and (16) presence of control group. Further, stressor-related information was extracted, including: (17) stressor type, (18) type of stress assessment, (19) manipulation check for stress induction (in case of experimental stressor), and (20) amount of stressor presentation. Extracted data regarding miRNAs contained: (21) examined miRNAs, (22) fluid collection for miRNA measures, (23) if participants fasted prior to miRNA collection, (24) amount of miRNA measurement, (25) time of miRNA collection, (26) storage temperature of miRNA samples, (27) storage duration of samples until miRNA analyses, and (28) miRNA analyses. Finally, data on results was extracted: (29) main methods, (30) main results and (31) confounding factors. Data extraction regarding miRNA analyses was cross checked by AB.

### 3.3 Quality assessment

To assess the risk of bias of included studies, we used the quality appraisal tool for biomarker-based cross-sectional studies (BIOCROSS) [68] for cross-sectional studies (n_studies_ = 4) (Table 1). The BIOCROSS tool consists of ten items (each with three issues to consider) within five domains. All items can be rated between two (all issues considered) and zero points (no issues considered), leading to a maximum score of 20 points. The tool was selected for its good inter-rater reliability and emphasis on biomarker investigation [68]. Moreover, it has been previously employed in studies investigating stress responses [e.g., Ref. 69].

**Table 1:** Risk of bias assessment of cross-sectional studies (all domains) and quasi-experimental studies (domain 5) with the BIOCROSS tool [68].

BIOCROSS											
	Domain 1 Study Rational	Domain 2 Design/Methods		Domain 3 Data Analysis		Domain 4 Data Interpretation		Domain 5 Biomarker Measurement			Overall
	Item 1	Item 2	Item 3	Item 4	Item 5	Item 6	Item 7	Item 8	Item 9	Item 10	
**Cross-sectional (all domains)**											
Chiba et al. (2022) [60]	2	2	1	1	1	2	1	1	0	1	**12 (fair)**
Holliday et al. (2025) [58]	2	1	1	2	1	2	1	2	1	0	**13 (fair)**
Krammer et al. (2023) [64]	2	1	0	2	2	2	1	1	0	0	**11 (fair)**
Pacheco et al. (2025) [65]	1	1	1	2	2	1	2	1	0	2	**13 (fair)**
**Quasi-experimental (domain 5 only)**											
Beech et al. (2014) [59]	-	-	-	-	-	-	-	1	1	1	**3 (fair)**
Gidron et al. (2010) [61]	-	-	-	-	-	-	-	1	1	1	**3 (fair)**
Honda et al. (2013) [62]	-	-	-	-	-	-	-	1	1	0	**2 (poor)**
Jurkiewicz et al. (2021) [63]	-	-	-	-	-	-	-	1	1	1	**3 (fair)**
Katsuura et al. (2012) [56]	-	-	-	-	-	-	-	1	0	1	**2 (poor)**
Maffioletti et al. (2021)^a^ [57]	-	-	-	-	-	-	-	2	1	1	**4 (fair)**
Vaisvaser et al. (2016) [66]	-	-	-	-	-	-	-	1	1	1	**3 (fair)**
Wiegand et al. (2018) [67]	-	-	-	-	-	-	-	2	1	1	**4 (fair)**

Note. Studies are listed in alphabetic order (first author).

a Quality was assessed only for baseline measurements; not for longitudinal study section which was not considered within this review.

The risk of bias of the remaining studies (n_studies_ = 8) was assessed using the revised JBI critical appraisal tool for quasi-experimental studies [70] (Table 2). The previous version of this tool was recommended [71]. It evaluates internal and statistical conclusion validity. Each question can be rated as yes (met), no (unmet), unclear or not applicable [70]. In line with a previously conducted stress-related systematic review and meta-analysis [69], we supplemented the JBI critical appraisal tool for quasi-experimental studies with domain five of the BIOCROSS tool [68] to specifically assess the quality of biomarker measurement. This combined approach allowed for a more comprehensive evaluation of both study validity and biomarker-related methodological rigor. AB and NR independently assessed the methodological quality of all included studies. Conflicts were resolved through discussion between AB and NR first. If no agreement was reached, AMA and PH were consulted.

**Table 2:** Risk of bias assessment of quasi experimental studies with the revised JBI critical appraisal tool for quasi-experimental studies [70].

Revised JBI CRITICAL APPRAISAL TOOL FOR QUASI-EXPERIMENTAL STUDIES										Overall
Internal validity bias related to:									Statistical conclusion validity^c^	(% YES)
	Temporal precedence	Selection and allocation	Confounding factors	Admin. of intervention/exposure	Assessment, detection, and measurement of the outcome			Participant retention		
	1	2	3	4	5^a^	6^a^	7^a^	8^a^	9^a^	
Beech et al. (2014) [59]	YES	NO	YES	YES	YES	YES	N.A.	N.A.	YES	**66.7%**
Gidron et al. (2010) [61]	YES	NO	YES	NO	YES	YES	N.A.	N.A.	NO	**44.4%**
Honda et al. (2013) [62]	YES	NO	YES	NO	NO	YES	N.A.	N.A.	YES	**44.4%**
Jurkiewicz et al. (2021) [63]	YES	NO	YES	YES	YES	YES	N.A.	N.A.	NO	**55.6%**
Katsuura et al. (2012) [56]	YES	NO	YES	NO	NO	YES	N.A.	N.A.	NO	**33.3%**
Maffioletti et al. (2021)^b^ [57]	YES	NO	YES	NO	NO	YES	N.A.	N.A.	YES	**44.4%**
Vaisvaser et al. (2016) [66]	YES	NO	YES	YES	YES	YES	N.A.	N.A.	NO	**55.6%**
Wiegand et al. (2018) [67]	YES	NO	YES	YES	YES	YES	N.A.	N.A.	YES	**66.7%**

Note. Studies are listed in alphabetic order (first author). Abbreviations: Admin.: Administration; N.A.: Not applicable.

a We refrained from reporting questions 5–9 separately for each outcome and result and focused solely on the outcome relevant to this review.

b Quality was assessed only for baseline measurements; not for longitudinal study section which was not considered within this review.

c Item nine was rated with ‘YES’, if three out of five conditions (test assumptions, G*Power, effect sizes, statistical methods, confounding factors) were given.

In accordance with a previous review and meta-analysis [69], we classified article quality as poor (<50% criteria met), fair (50% - 75% criteria met), or good (≥75% criteria met), based on BIOCROSS ratings. For the revised JBI tool, quality was classified based on the number of ‘Yes’ responses. No thresholds were defined; and no specific weight was assigned to any domain. According to the general recommendation from JBI in quantitative reviews, we did not exclude any studies based on our quality assessment [70]. Although quality of each study should be considered when interpreting the results, by including all studies we aim to reduce bias through selective evidence.

## 4 Results

### 4.1 Methodological results

#### 4.1.1 Participant characteristics

In most studies, participants were recruited in the United States [58,59,63,65], followed by Japan [56,60,62], Germany [61,67], Austria [64], Israel [66] and Italy [57]. The sample sizes varied from N = 10 [56] to N = 173 participants [64] with N = 489 participants included in total in this review. The age of the participants ranged from 18 to 84 years [64] with a weighted mean age of M = 42.9 years (only studies reporting data on mean age were considered [[56], [57], [58], [59], [60], [61], [62], [63], [64], [65]]). A balanced sex ratio of 50% (between male and female participants) was reported in three studies [56,65,67]. Of the N = 489 participants included in total, 52.5% were women (n = 257) No non-binary participants were included. Most participants were considered healthy [56,[60], [61], [62], [63],^66^,^67^], while one study included both healthy participants and those with alcohol abuse or binge drinking (but not alcohol dependence) [59]. Another study included participants with and without stress-related diseases [64], while a further study examined participants with treatment-resistant depression, some of whom had comorbidities such as PTSD or psychotic symptoms [57]. Five studies did not report data on medication intake [58,61,[64], [65], [66]]. During one interventional study, participants received pharmacological treatment [57], but only baseline data is being considered in this review. Five studies did not include participants taking medication [56,59,60,62,67], while another study excluded participants on specific types of medication, e.g. hormonal or cardiovascular-related medication [63]. Due to the limited number of studies and inconsistent reporting of data across studies, we did not conduct subgroup or sensitivity analyses on the medication intake or health status of the participants. Three studies excluded participants on oral contraceptives or reported that no participants used oral contraceptives [56,59,67] while one study accounted for the menstruation cycle phase [67]. One study examined breastfeeding women 30 days after delivery [60]. Assessment of participants’ exercise levels was reported within two studies [61,64]. Two studies [60,66] included not only human but also animal data (mice); nevertheless, these study sections are beyond the scope of this review and are not considered here.

In six studies, no human control group [60] or a pre-post design with participants serving as their own controls (within-subject design) was implemented [56,[61], [62], [63],^67^]. Although not directly focused on stress, one study compared heavy and moderate alcohol drinkers [59]. One study compared therapy responders and non-responders. However, the longitudinal intervention component of the study was not included in this review; only baseline stress assessments are reported, since therapy response falls outside the scope of this review [57]. Four studies compared groups based on their stress levels, with distinct categorizations. Out of those, two studies compared low vs. high stress groups based on self-reported data [58,64]. Another study compared low vs. high perceived discrimination (based on self-reported questionnaire data) and participant characteristics (African American vs. White women) [65], while one study compared two groups based on their self-reported stress levels after experimental stress induction (stress sustainment vs. recovery) [66]. Table 3 presents detailed participants’ characteristics.

**Table 3:** Participant characteristics.

First Author (year)	N	Age (year) M ± SD [min. – max.]	Sex female n (%)	BMI M ± SD [min. – max.]	Smoking status	Health status
Beech et al. (2014) [59]	22	29.2^a^ ± n.a. [21-50]	6 (27.3%)	n.a.	Non-smoker	Healthy/alc. abuse/binge drinking
Chiba et al. (2022) [60]	25	31.1 ± 1.1 [21-39]	25 (100%)	20.9 ± 0.6 [18.0 - 24.5]	Non-smoker	Healthy
Gidron et al. (2010) [61]	37	22.7 ± 2.2 [n.a.]	28 (75.7%)	n.a.	n.a.	Healthy
Holliday et al. (2025) [58]	12	19.7 ± 0.3 [n.a.]	12 (100%)	n.a.	n.a.	n.a.
Honda et al. (2013) [62]	25	25.5 ± 0.4 [n.a.]	0 (0%)	21.8 ± 2.3 [n.a.]	Non-smoker	Healthy
Jurkiewicz et al. (2021) [63]	12^b^	43.2 ± 19.3 [n.a.]	0 (0%)	n.a.^c^	Non-smoker	Healthy
Katsuura et al. (2012) [56]	10	22.4 ± 0.8 [n.a.]	5 (50%)	22.5 ± 3.0 [18.8 - 26.3]	Non-smoker	Healthy
Krammer et al. (2023) [64]	173	49.7 ± 14.4 [18-84]	110 (63.6%)	25.4 ± 5.1 [17.3 - 46.8]	n.a.	Subgroup with stress-related disorders
Maffioletti et al. (2021) [57]	41^d^	52.1 ± 8.7 [n.a.]	29 (70.7%)	n.a.	Smoker and Non-smoker	Treatment-resistant major depressive disorder
Pacheco et al. (2025) [65]	59	54.3 ± 5.6^e^ [45-64]	30 (50%)^f^	n.a.	n.a.	n.a.
Vaisvaser et al. (2016) [66]	49	n.a. [19-22]	0 (0%)	n.a.	n.a.	Healthy
Wiegand et al. (2018) [67]	24	n.a. [18-35]	12 (50%)	n.a.	Non-smoker	Healthy

Note. Studies are listed in alphabetic order (first author). All values have been rounded to one decimal place.

Abbreviations: alc.: alcohol; BMI: Body Mass Index; max.: maximum; M: mean, min.: minimum; N: sample size; n.a.: data not available; SD: standard deviation.

a Only average age given in publication.

b N = 55 participants included in study; miRNA analyses for n = 12 participants available only.

c BMI ≥30 as study exclusion.

d Data on miRNA levels correlating with the Holmes-Rahe Life Stress Inventory [72] available for n = 33 participants only.

e Weighted M and pooled SD were calculated manually from subgroup data available.

f Age and sex data were available for N = 60 participants, whereas miRNA data were available for n = 59 participants only.

#### 4.1.2 Psychosocial stress evaluation and study characteristics

Different methodological approaches were used to induce psychosocial stress in participants. Three studies used an academic/naturalistic stressor (exam). All those studies assessed stress subjectively via self-report questionnaires and confirmed a significant change in stress experience in relation to the academic stressor [56,61,62]. Two of those studies additionally measured biological stress-markers [56,62]. One study examined salivary cortisol, showing significantly higher cortisol levels two months pre compared to one month post stressor. However, cortisol levels two days prior to the stressor did not change significantly [62]. In contrast, one study reported significant changes in salivary cortisol and cytokine levels one day before or immediately after the stressor compared to one week post stressor [56]. All three studies measured miRNA expression multiple times (two to four measurement points) over weeks/months. It should be considered that two studies assessed baseline measures one week or one month after stress exposure rather than prior to the examination. The temporal alignment of pre- and post-measurements is therefore limited [56,62].

Four studies used an laboratory paradigm [59,63,66,67], most commonly the original or modified version of the TSST [63,66,67]. All those studies assessed stress subjectively (self-report questionnaire) and objectively using biological stress markers with partially distinct results. For instance, one study selected participants for miRNA analysis based on a predefined criterion of a robust salivary cortisol response to the TSST [63]. In contrast, another study found no significant change in cortisol over time but did observe changes in heart rate and stress ratings [66]. One study reported changes in heart rate variability, salivary amylase, cortisol and stress ratings in response to the stressor [67]. All four studies measured miRNA expression at multiple times (arrival to 3 hours post stress induction) within one day [59,63,66,67].

Moreover, five studies relied on participant self-reports to assess subjective stress levels through interviews or questionnaires [57,58,60,64,65]. Two studies assessed stress within the past 12 months [57,58], while one study considered lifetime experiences [65]. In contrast, one study assessed current stress [64], while one study did not specify the stress period considered [60]. None of the studies measured biological markers of stress, except of one study assessing CRP as a marker of inflammation in response to psychotherapy [57]. Additional details are provided in Table 4. Further information on miRNA collection and storage is given in Chapter 4.1.3.

**Table 4:** Psychosocial stress induction, assessment and miRNA sampling timepoints.

Authors (year)	Stress induction	Psychological stress assessment	Biological stress assessment	Period of stress evaluation (miRNA sampling points)
	**Academic stressor**			
Gidron et al. (2010) [61]	University exam (medicine)	PSQ [73]	None	Post semester break -post stressor (x2)
Honda et al. (2013) [62]	National medical license examination	STAI-S [74]	Cortisol (saliva)	2 months pre – 1 month post stressor (x3)
Katsuura et al. (2012) [56]	Nationally administered examination for academic promotion (medicine)	STAI-S [74]	Cortisol (saliva), IL-1β, IL-6, TNF-α, IFN-γ (serum)	7 weeks pre – 1 week post stressor (x4)
	**Laboratory stressor**			

Beech et al. (2014) [59]	Personalized stressful imagery task	Mood rating (not further specified)	ACTH (blood), cortisol (blood), basal norepinephrine (blood), blood pressure, HR	1h pre – 1h post stressor (x3)
Jurkiewicz et al. (2021) [63]	TSST [13]	VAS [75] on perceived stress; MDMQ^a^ [76]	Cortisol (saliva)	Arrival – 105 min post stressor (x3)
Vaisvaser et al. (2016) [66]	Arithmetic task (TSST modified) [13]	Nine-point Likert scale on stress	Cortisol (saliva), HR	Arrival – 3h post stressor (x2)
Wiegand et al. (2018) [67]	TSST (modified) [13,77]	VAS [75] on perceived stress	Cortisol (saliva), alpha-amylase (saliva), HR, HRV	10 min pre stressor – 1h post stressor (x4)
	**No stress induction**			

Chiba et al. (2022) [60]	None	POMS-2 short version [78]	None	n.a. (x1)
Holliday et al. (2025) [58]	None	SRSS [79]	None	Past year (x3 (morning – afternoon))
Krammer et al. (2023) [64]	None	Interview on stress levels and stress-related diseases (not further specified)	None	Current (x1)
Maffioletti et al. (2021) [57]	None	Paykel Scale of stressful life events [72]; SRSS [79]	CRP (as measurement of inflammation)	Past 12 months (x4^b^)
Pacheco et al. (2025) [65]	None	Questionnaire on Lifetime discrimination [80] and racial discrimination [81]	None	Lifetime (x1)

Note. Abbreviations: ACTH: Adrenocorticotropic hormone; CRP: C-reactive protein; h: hour; HR: heart rate; HRV: heart rate variability; IFN = Interferon; IL = Interleukin; MDMQ: Multidimensional Mood State Questionnaire [76]; min.: minutes; n.a.: no data available; POMS-2: Profile of Mood State Second Edition-Adult [78]; PSQ: Perceived Stress Questionnaire [73]; SRSS: Holmes and Rahe Social Readjustment Scale [79]; STAI-S: State-Trate Anxiety Inventory [74]; TNF = Tumor Necrosis Factor; TSST: Trier Social Stress Test [13]; VAS: Visual Analogue Scale [75].

a Received information from author (AMA).

b only baseline measurement (x1) considered in this review.

#### 4.1.3 miRNA collection and storage

The amount of miRNA measurements varied from one [60,64,65] to four time points [56,67]. One study collected miRNAs at three times in one day to normalize circadian biological alterations [58]. One study assessed miRNA alterations over four measurement time points, however only baseline assessment was considered in this review [57]. Most studies analyzed miRNA using blood samples [56,57,59,[61], [62], [63], [64], [65], [66]]. Two studies used whole blood samples [56,59], three studies assessed miRNAs in Peripheral Blood Mononuclear Cells (PBMC) [63,65,66], in one study capillary blood [64] and in two studies peripheral venous blood samples were collected [57,62]. In one study no information was provided on the blood samples withdrawn [61]. Two studies extracted miRNAs from saliva [58,67], while one study extracted miRNA from breast milk [60]. Four studies did not report the time of miRNA collection [61,[64], [65], [66]], while five studies assessed miRNAs in the afternoon until evening [56,59,62,63,67] and two studies during (mid-) morning [57,60]. Most studies did not report whether participants were required to fast prior to sample collection for miRNA analysis [56,58,[60], [61], [62], [63], [64],^66^,^67^], while three studies collected samples following a fasting period [57,63,65] in one study, participants had a standard lunch 3 h prior to stress exposure and consumed beer shortly after, still during sample collection period [59]. Overall, the samples were ultimately stored frozen at –80°C in most of the studies [[56], [57], [58], [59], [60],^62^,^63^,[65], [66], [67]], while one study stored their samples at –20°C before miRNA analyses [64], although some samples were kept at higher temperatures temporarily before storage. One study did not report storage temperature until subjected to miRNA isolation [61].

#### 4.1.4 miRNA isolation and analyses

Most studies did not report storage duration of samples until miRNA isolation or analyses [57,[59], [60], [61],[63], [64], [65], [66], [67]]. Two studies reported the time period of (mi)RNA isolation from collected samples lasting from same day [58] to within two weeks after collection [56], while duration until further analyses was not given.

Commercially available miRNA extraction kits were used to isolate miRNA [61,63]. Subsequently, nanodrop or similar methods were applied to assess the quality and amount of isolated miRNA prior to subjecting them for miRNA specific (low-throughput) e.g., Refs. [[59], [60], [61],^64^,^67^] or genome-wide quantification (high-throughput) e.g., Refs. [56,62,63,65,66]. Six studies investigated a small amount of specific pre-defined miRNAs [57,[59], [60], [61],^64^,^67^], while five studies investigated a substantial number of miRNAs or followed a genome-wide approach [56,62,63,65,66]. Six studies solely used RT-qPCR based assays to quantify miRNAs [[59], [60], [61],^64^,^66^,^67^], however, others used high-throughput methods like microarray profiling [56,62,63] and Illumina sequencing [65]. All high-throughput studies have additionally confirmed their respective target miRs by qPCR, to nullify risk of false-positives. All studies provided the results after targeted statistical analysis.

### 4.2 miRNA analysis results

#### 4.2.1 miRNA expression in response to psychosocial stress

All included studies reported significant stress-associated changes in miRNA expression in multiple body fluids (blood, saliva, breast milk) in response to various stressors (academic, laboratory) and in association with self-reported questionnaire and interview data on stress exposure as well as over different observation periods (immediately post stressor – lifetime stress experiences) (see Table 4). A total of 34 differentially expressed miRNAs were identified. Six miRNAs (17.7%) (miR-10a-5p; miR-16; miR-20b; miR-21; miR-26b; miR-144-3p/-5p) were identified as significantly differentially expressed in more than one study and are discussed separately in section 4.2.5. Most altered miRNAs were identified in blood samples (n_miR_ = 35, 81.4%), followed by saliva (n_miR_ = 7, 16.3%), and breast milk (n_miR_ = 1, 2.3%). Our results show both increases as well as decreases in miRNA expression in response to psychosocial stress. In the following, the results are presented according to psychosocial stressor type; however given the considerable variability in methods and observation periods, all results should be interpreted and compared with caution. Furthermore, we would like to point out that miRNA nomenclature has evolved over time [82] and was not applied uniformly across the studies included in this review. Bias and errors may arise when comparing the results across studies, due to inconsistent naming. To address this issue, we have supplemented Table 5 with the miRNA nomenclature reported in the respective studies and, where possible, included information on how the miRNA nomenclature (-3p/-5p strand) was harmonized. The spelling of ‘miR’ (e.g., italicized, with uppercase R and hyphens) was adopted from the spelling used in the publications included here, even though the current guidelines do recommend slightly different spelling in some case [82].

#### 4.2.2 miRNA expression in response to academic stressors

Of the 28 altered miRNAs identified in one study only, three (10.7%) (let-7b, miR-29a, miR-126) exhibited differential expression in response to academic stress in two separate studies [61,62]. All miRNAs were identified in blood samples. Two miRNAs showed elevated levels prior to a National Medical License Examination, compared to one month after the examination (baseline) [62]. In contrast, one miRNA showed decreased expression in response to a university exam compared to levels observed after the semester break [61].

#### 4.2.3 miRNA expression in response to laboratory stressors

Of the 28 altered miRNAs identified in a single study only, 15 (53.6%) (miR-9-5p, miR-29c, miR-92a-2-3p, miR-129-5p, miR-137-3p, miR-210-3p, miR-301b, miR-375-3p, miR-518a-2, miR-614, miR-624-3p, miR-937-3p, miR-1185-5p, miR-3178, miR-3191-3p) showed altered expression in response to laboratory stressors in two studies that both used the TSST [63] or a modified version of the TSST [66] to elicit acute psychosocial stress. All miRNAs were identified in blood samples. Significantly decreased expression was observed in eight of the miRNAs 45 or 105 min following psychosocial stress induction using the TSST compared to baseline or 45 min post TSST [63]. However, three miRNAs exhibited increased expression 45 or 105 min following the TSST compared to baseline or 45 min post TSST [63]. Notable, three miRNAs exhibited a similar pattern: their expression significantly decreased 45 min after TSST compared to baseline, followed by a significant increase 105 min post TSST exposure [63]. In contrast, miR-29c showed participant-specific differences, with nearly half of the participants exhibiting either decreased or increased expression following a modified TSST task [66].

#### 4.2.4 miRNA expression in association with self-reported stress

Of the 28 altered miRNAs reported in only one study, ten (35.7%) (let-7a-5p, let-7g-5p, miR-15a-5p, miR-19b-3p, miR-34a-3p, miR-135-3p, miR-146a-5p, miR-148a-3p, *MIR155HG*, miR-187-3p) showed differential expression in association with self-reports of stress experience based on questionnaires or interviews in five studies [57,58,60,64,65]. It is important to note that *MIR155HG* is considered a host gene encoding miR-155-3p/-5p, therefore direct comparison with the other miRNAs reported here might be challenging. Most of the miRNAs were identified in blood samples [57,64,65], while one study identified miRNAs in saliva [58] followed by the identification of one altered miRNA in breast milk [60]. Five miRNAs showed decreased expression in association with questionnaire-based assessments of stressful life events in the past 12 months [57,58] and mood disturbances assessed by questionnaires [60]. One study reported differential expression of *MIR155HG,* not in relation to stress levels, but in African American women reporting high discrimination compared to White women also reporting high discrimination [65]. Additionally, four miRNAs showed increased expression in association with stressful life events that occurred during the previous 12 months of assessment [58], as well as with an interview-based self-report of current stress levels and stress-related diseases [64].

#### 4.2.5 Altered stress-associated miRNA expression identified in more than one study

Of the six miRNAs that demonstrated altered stress-associated expression in multiple studies (miR-10a-5p, miR-16, miR-20b, miR-21, miR-26b, miR-144-3p/-5p) most were investigated across two studies [56,59,62,64,67] while miR-21 showed differential expression across three studies [59,61,67]. Three miRNAs (miR-10a-5p, miR-16, miR-144-3p/-5p) were observed in the same body fluid (blood), while three miRNAs (miR-20b, miR-21, miR-26b) were examined in blood and saliva samples. Increased expression of miR-10a-5p was observed in two studies in response to a laboratory stressor (stress imagery task) [59] and in association with self-reported current stress [64]. Increased miR-16 and miR-144-3p/-5p levels were reported in two studies each in response to an academic stressor [56,62]. Altered expression (increases and decreases) of miR-20b and miR-26b was reported in two studies involving laboratory (modified TSST) [67] and academic stressors [62]. Differential expressions of miR-21 (increase and decreases) were observed in three studies using a laboratory or academic stressor to induce psychosocial stress (TSST; stress imagery task; exam) [59,61,67]. Table 5 provides further information on those miRNAs found to be stress-associated differentially expressed in numeric order.

**Table 5:** Differentially expressed miRNAs in response to psychosocial stress, sorted by biological sample and hsa-miR in numerical order.

miRNA	Body fluid							Altered stress-related miRNA expression
	Blood				Breast milk	Saliva		
	whole	PBMC	Capi.	Peri.				
hsa-let-7a-5p^a^			x				↑	Significant differences between stress and control group; self-reported stress (interview) as significant predictor of let-7a-5p expression (*p* = 0.000); stressed participants had 0.653 **higher expression** of let-7a-5p vs. control group (ANCOVA, multiple linear regression analyses) [63]
hsa-let-7b^c^	x*						↓	let-7b levels significantly **declined** between low (baseline) and high stress (after exam) t(36) =2.15, *p* = 0.04 (2-tailed) (paired *t*-test) [60]
hsa-let-7g-5p^a^			x				↑	Significant differences between stress and control group; self-reported stress (interview) as significant predictor of let-7g-5p expression (*p* = 0.006); stressed participants had 0.362 **higher expression** of let-7g-5p vs. control group (ANCOVA, multiple linear regression analyses) [63]
hsa-miR-9-5p^a^		x					↓	Significantly **decreased** miR-9-5p expression 105 min post TSST vs. baseline (|FC| -1.3) and 105 min. vs. 45 min. post TSST (|FC| -1.2), *p* < 0.01 (linear models) [62]
**hsa-miR-10a-5p**^a^^,^^b^	x						↑	**Increased** miR-10a-5p **expression** one hour post stress induction in heavy (fold induction = 1.24 ± 0.16), not in moderate alcohol drinking participants (fold induction = 1.05 ± 0.15); group differences not significant (MANOVA for repeated measures) [58]
			x				↑	Significant differences between stress and control group; self-reported stress (interview) as significant predictor of miR-10a-5p expression (*p* = 0.020); stressed participants had 0.594 **higher expression** of miR-10a-5p vs. control group (ANCOVA, multiple linear regression analyses) [63]
hsa-miR-15a-5p^a^			x				↑	Significant differences between stress and control group; self-reported stress (interview) as significant predictor of miR-15a-5p expression (*p* = 0.040); stressed participants had 0.279 **higher expression** of miR-15a-5p vs. controls (ANCOVA, multiple linear regression analyses) [63]
**hsa-miR-16**^b^^,^^c^				x			↑	Significantly **higher** miR-16 levels two months and two days pre compared to one month post examination (F(2,48) = 7.155, *p* = 0.0019, η_p_^2^ = 0.86) (repeated measures ANOVA) [61]
	x						↑	miR-16-5p levels significantly **increased** from seven weeks prior to immediately post examination (F(3,27) = 3.791, *p* = 0.022) (only when normalized by RNU48 (repeated measures ANOVA) [55]
hsa-miR-19b-3p^a^						x	↓	Significant **downregulation** of miR-19b-3p in high chronically stressed participants vs. low chronically stressed participants, *x*^2^(2, N=12) = 7.42, *p* < 0.01, η^2^ = 0.915 (Kruskal-Wallis test) [57]
**hsa-miR-20b**^a^^,^^c^				x			↑	Significantly **higher** miR-20b levels two months and two days pre compared to one month post examination (F(2,48) = 8.256, *p* = 0.0008, η_p_^2^ = 0.90) (repeated measures ANOVA) [61]
						x	↑↓↑	Median miR-20b-5p expression **increased** from baseline (ΔCT = 2.74) to end of TSST (ΔCT = 2.11), **decreased** 30 min post TSST (ΔCT = 2.74), **increased** 60 min post TSST (ΔCT = 1.85), *p* < 0.05, *d* = 0.7103 (Friedman test for repeated measures) [66]
**hsa-miR-21**^a^^,^^b^^,^^c^	x						↑	**Increased** miR-21-5p expression one hour post stress induction in heavy (fold induction = 1.59 ± 0.32), not in moderate alcohol drinking participants (fold induction = 1.01 ± 0.24); group differences not significant (MANOVA for repeated measures) [58]
	x*						↓	miR-21 levels significantly **declined** between low (baseline) and high stress (after exam) t(36)=2.69, *p* = 0.01 (2-tailed) (paired *t*-test) [60]
						x	↓	Median miR-21-5p expression was significantly **higher** at baseline (ΔCT = -0.82) and end of TSST (ΔCT = -0.39) vs. 30 min. (ΔCT = 0.57) and 60 min post TSST (ΔCT = 0.67). Expression **decreased** from baseline to 60 min post TSST with strongest decrease after TSST, *p* < 0.05, *d* = 0.8563 (Friedman test for repeated measures) [66]
**hsa-miR-26b**^a^^,^^c^				x			↑	Significantly **higher** miR-26b levels two months and two days pre compared to one month post examination (F(2,48) = 11.61, *p* = 0.0001, η_p_^2^ = 0.86) (repeated measures ANOVA) [61]
						x	↑↓	Median-26b-5p expression **increased** from baseline (ΔCT = 0.85) to end of TSST (ΔCT = 0.11) and **decreased** 30 min post TSST (ΔCT = 1.46, *p* < 0.001, *d* = 1.1236 (Skillings-Mack test) [66]
hsa-miR-29a^c^				x			↑	Significantly **higher** miR-29a levels two days pre compared to one month post examination (F(2,38) = 3.880*, p* = 0.0293, η_p_^2^ = 0.95) (repeated measures ANOVA) [61]
hsa-miR-29c^c^		x					↓↑	n = 27 participants showed a **decrease** in miR-29c expression FC (mean ± SD): 0.73 ± 0.18, n = 22 showed an **increase** in expression FC (mean ± SD) 1.44 ± 0.32 post arithmetic task (TSST modified); greater miR-29c expression changes in the stress sustainment vs. stress recovery group (F(1,47) = 6.50, *p* = 0.014, *d* = 0.72 (ANOVA) [65]
hsa-miR-34a-3p^a^						x	↓	Significant **downregulation** of miR-34a-3p in high chronically stressed participants vs. low chronically stressed participants, *x*^2^(2, N=12) = 7.42, *p* < 0.05, η^2^ = 0.60 (Kruskal-Wallis test) [57]
hsa-miR-92a-2-3p^a^		x					↓↑	**Significantly decreased** miR-92a-2-3p expression 45 min post TSST vs. baseline (|FC| -1.2), *p* < 0.01, significantly **increase** in expression 105 min. vs. 45 min. post TSST (|FC| 1.2), *p* < 0.01 (linear models) [62]
hsa-miR-126^c^				x			↑	Significantly **higher** miR-126 levels two months and two days pre compared to one month post examination (F(2,48) = 6.800, *p* = 0.0025, η_p_^2^ = 0.98) (repeated measures ANOVA) [61]
hsa-miR-129-5p^a^		x					↑	**Increased** miR-129-5p expression 45 min post TSST vs. baseline (|FC| 1.2), *p* < 0.01 (linear models) [62]
hsa-miR-135-3p^a^						x	↑	Significant **upregulation** of miR-135-3p in high chronically stressed vs. low chronically stressed participants, *x*^2^(2, N=12) = 8.00, *p* < 0.05, η^2^ = 0.67 (Kruskal-Wallis test) [57]
hsa-miR-137-3p^a^		x					↓	**Decreased** miR-137-3p expression 45 min post TSST vs. baseline (|FC| -1.2), *p* < 0.01 (linear models) [62]
**hsa-miR-144-3p/-5p**^d^				x			↑	Significantly **higher** miR-144-3p levels two months and two days pre compared to one month post examination (F(2,48) = 9.138, *p* = 0.0004, η_p_^2^ = 0.91) (repeated measures ANOVA) [61]
	x						↑	miR-144-3p levels significantly **increased** immediately post examination vs. seven weeks pre and one week post examination (F(3,27) = 4.614, *p* = 0.010) (when normalized by RNU48); (F(3,27) = 4.241, *p* = 0.014) (when normalized by U6 snRNA) (repeated measures ANOVA) [55]
				x			↑	Significantly **higher** miR-144-5p levels two months and two days pre compared to one month post examination (F(2,48) = 11.80, *p* < 0.0001, η_p_^2^ = 0.97) (repeated measures ANOVA) [61]
	x						↑	Significantly **increased** miR-144-5p levels from seven weeks prior to immediately post examination (F(3,27) = 4.115, *p* = 0.016) (when normalized by RNU48); (F(3,27) = 3.743, *p* = 0.023) (when normalized by U6 snRNA) (repeated measures ANOVA) [55]
hsa-miR-146a-5p^b^				x			↓	Significant **negative correlation** between miR-146a-5p expression and recent stressful life events (Holmes scale: *p* = 0.001, t = −0.366 (Kendall rank coefficient) [56]
hsa-miR-148a-3p^a^					x		↓	Relative miR-148a-3p expression was significantly **negatively correlated** with the total mood disturbance T-score (POMS-2), Spearman *r* = -0.683, 95% CI [-0.767, -0.174]. Each negative mood subscale (T-score) was significantly negatively correlated with relative miR-148a-3p expression (*r* = -0.485, 95% CI [-0.651 – 0.058] – *r* = -0.722, 95% CI [-0.686, -0.001]). Positive mood subscales (T-score) were significantly positively correlated with relative miR-148a expression (*r* = 0.465, 95% CI [-0.029, 0.668] and *r* = 0.538, 95% CI [0.174, 0.767] (Spearman rank correlation coefficient) [59]
*hsa-MIR155HG*^e^		x					↓	*MIR155HG* significantly **decreased** in African American women with highly experienced discrimination compared to White women with high discrimination (log_2_|FC| -2.5466), *padjusted* = 0.0358 (DESeq2 version 1.30.1) [64]
hsa-miR-187-3p^a^						x	↓	Significant **downregulation** of miR-187-3p in high chronically stressed vs. low chronically stressed participants, *x*^2^(2, N=12) = 7.36, *p* < 0.05, η^2^ = .598 (Kruskal-Wallis test) [57]
hsa-miR-210-3p^a^		x					↓	Significantly **decreased** miR-210-3p expression 105 min post TSST vs. baseline and 105 min. vs. 45 min. post TSST (|FC| -1.3), *p* < 0.01 (linear models) [62]
hsa-miR-301b^c^		x					↓	Significantly **decreased** miR-301b expression 105 min. vs. 45 min. post stressor (|FC| -1.2), *p* < 0.01 (linear models) [62]
hsa-miR-375-3p^a^		x					↓	Significantly **decreased** miR-375-3p expression 105 min. vs. 45 min. post TSST (|FC| -1.2), *p* < 0.01 (linear models) [62]
hsa-miR-518a-2^c^		x					↓↑	Significantly **decreased** miR-518a-2 expression 45 min post TSST vs. baseline (|FC| -1.2), *p* < 0.01; significantly **increased** expression 105 min. vs. 45 min. post TSST (|FC| 1.2), *p* < 0.01 (linear models) [62]
hsa-miR-614^c^		x					↓↑	Significant **decrease** in miR-614 expression 45 min post TSST vs. baseline (|FC| -1.2), *p* < 0.01; significant **increase** 105 min. vs. 45 min. post TSST (|FC| 1.2), *p* < 0.01 (linear models) [62]
hsa-miR-624-3p^a^		x					↑	Significantly **increased** miR-624-3p expression 105 min post TSST vs. baseline (|FC| 1.2) and 105 min. vs. 45 min. post TSST (|FC| 1.3), *p* < 0.01 (linear models) [62]
hsa-miR-937-3p^a^		x					↑	Significantly **increased** miR-937-3p expression 105 min post TSST vs. baseline (|FC| 1.2), *p* < 0.01 (linear models) [62]
hsa-miR-1185-5p^a^		x					↓	Significant **decrease** of miR-1185-5p expression 105 min. vs. 45 min. post TSST (|FC| -1.2), *p* < 0.01 (linear models) [62]
hsa-miR-3178^c^		x					↓	Significant **decrease** in miR-3178 expression 45 min (|FC| -1.2) and 105 min (|FC| -1.2) post TSST vs. baseline, *p* < 0.01 (linear models) [62]
hsa-miR-3191-3p^a^		x					↓	Significant **decrease** in miR-3191-3p expression 105 min. vs. 45 min. post TSST (|FC| -1.2), *p* < 0.01 (linear models) [62]

Note. Abbreviations: ANOVA: analysis of variance; capi.: capillary blood; FC: fold change; hsa: homo sapiens; MANOVA: multivariate analysis of variance; miR: microRNA; min.: minute; PBMC: Peripheral Blood Mononuclear Cell; Peri.: peripheral blood; SD: standard deviation; whole: whole blood; ↑ = increases expression; ↓ = decreased expression.

x* = not further specified.

*miR-877-5p* was identified by Krammer et al., 2023 [63] as being significantly higher expressed in stressed participants vs. controls (independent *t*-test); however, this association did not remain significant after adjusting for the covariates age and BMI (ANCOVA) and was therefore not included here.

*miR-26b, -126, -15a, -19a, -106b, -142-3p, -19b and -30b* were identified by Katsuura et al., 2012 [55] as significantly differentially expressed in response to psychosocial stress (repeated measures ANOVA without multiple testing correction). However, this did not remain significant after repeated measures ANOVA with multiple testing correction and after validation by qPCR and were therefore not included here.

miRNAs written in bold letters were identified in multiple studies.

a miR-3p/-5p strand was clearly given in publication.

b miR-3p/5p strand was identified with TaqMan assay ID and miRBase [[83], [84], [85], [86], [87], [88]]: (hsa-miR-10a-5p (TaqMan Assay ID: 000387) (https://www.mirbase.org/results/?query=MIMAT0000253) [[83], [84], [85], [86], [87], [88]] [58]; hsa-miR-16-5p (TaqMan Assay ID: 000391) (https://www.mirbase.org/results/?query=MIMAT0000069) [[83], [84], [85], [86], [87], [88]] [55]; hsa-miR-21-5p (TaqMan Assay ID: 000397) (https://www.mirbase.org/results/?query=MIMAT0000076) [[83], [84], [85], [86], [87], [88]] [58]; hsa-miR-146a-5p (TaqMan Assay ID: 000468) (https://www.mirbase.org/results/?query=MIMAT0000449) [[83], [84], [85], [86], [87], [88]] [56].

c strand not specified in publication.

d Originally reported in publications as hsa-miR-144* and hsa-miR-144. We adapted hsa-miR-144* to hsa-miR-144-5p and hsa-miR-144 to hsa-miR-144-3p according to miRBase (https://www.mirbase.org/results/?query=hsa-mir-144) [[83], [84], [85], [86], [87], [88]].

e *MIR155HG*: host gene, encoding miR-155-3p and miR-155-5p.

### 4.3 miRNA determinants and metabolic impact

#### 4.3.1 Factors associated with miRNA expression

Some studies reported here [57,59,61,62,64] identified factors associated with miRNA expression, for instance, state anxiety (miR-16) [62], and stress-related diseases like anxiety (let-7a-5p, let-7g-5p) [64], depression (let-7a-5p), chronic fatigue syndrome and headache/migraine (let-7a-5p, let-7g-5p, miR-15a-5p) [64]. Nutritional habits were also associated with miRNA expression, including meat consumption (let-7a-5p) and coffee intake (miR-15a-5p) [64]. miRNA expression 1 hour following a laboratory stressor was correlated with the quantity of alcohol (beer) consumed shortly thereafter in participants with heavy alcohol consumption (miR-10a, miR-21) [59]. Considering sociodemographic factors, age (let-7f, miR-29a) was found to be correlated with miRNA expression [57]. In terms of lifestyle factors, baseline health behavior (e.g., physical exercise, stress control) moderated stress-related miRNA expression (miR-21) [61]. Further correlations of miRNAs and markers of stress reaction are discussed in Chapter 5.3.

#### 4.3.2 Which role do differentially expressed miRNAs in response to psychosocial stress play in metabolism and metabolic diseases? (Exploratory investigation)

An exploratory objective of this review is to explore the associations between stress-related changes in miRNA expression and their potential roles in metabolism and metabolic diseases.

To obtain initial indication, all stress-associated differentially expressed miRNAs identified here were searched in July and December 2025 by NR within the miRBase database (http://www.mirbase.org) [[83], [84], [85], [86], [87], [88]] and the Human microRNA Disease Database (HMDD v.4.0) (https://www.cuilab.cn/hmdd) [89]. Of the 34 stress-associated altered miRNAs identified here, 26 (76.5%) identified as being associated with metabolic (associated) diseases such as diabetes mellitus and complications of diabetes (e.g., diabetic retinopathy), obesity, metabolic syndrome, liver and pancreatic disorders, lipid metabolism disorders, vitamin and mineral metabolism disorders, mitochondrial disorders or endocrine and hormonal disorders, according to the HMDD v.4.0 [89]. Importantly, these findings are not limited to human but also animal studies. Given the exploratory nature of this objective, only a brief non-systematic overview of associations between specific miRNAs and metabolic diseases in humans is provided. Further information is given in Supplementary Table S1, which lists associations with metabolic diseases for each miRNA, and in Supplementary Table S2, offering target genes of those miRNAs identified in more than one study here.

## 5 Discussion

This systematic review focuses on differential miRNA changes in various body fluids in response to different acute and chronic psychosocial stressors in adults. Factors associated with miRNA expression were reported and associations with metabolic disorders were presented in an exploratory manner.

### 5.1 Findings on altered miRNA expression in response to psychosocial stress

Our first objective was to identify differential miRNA expression in response to psychosocial stress in adults. We summarized 12 studies in which 34 miRNAs were identified that were altered in various body fluids (blood, breast milk, saliva) in response to acute and chronic psychosocial stressors. The results yielded heterogeneous findings with stress-associated up- and downregulation of miRNAs. The heterogeneity of methodological approaches (e.g., stress assessment), together with the fact that most of the identified miRNAs were detected in only one, not multiple studies, limits comparability across studies and makes it difficult to draw a generalizable, robust conclusion. Interestingly, some studies examined the same miRNAs but reported distinct results. For instance, no stress-associated alterations of miR-16-5p [64,67], miR-19b-3p, miR-21-5p and miR-26b-5p [64] expression were found in some studies, whereas these miRNAs were identified as differentially expressed in other studies reported here (see Table 5). Further, inconsistencies in miRNA nomenclature were observed, which may introduce bias and lead to errors when comparing results across studies, as different strands may be referred to, potentially corresponding to distinct targets and biological functions. Over half of the studies reported here did not followed a genome-wide approach, but tested single miRNAs [[57], [58], [59], [60], [61],^64^,^67^], potentially leading to an early bias [38]. Also, differences in statistical approaches, such as the use of fold change thresholds, may limit comparability across studies. While some studies reported only genes with a fold-change absolute value of ≥2 [65] or miRNAs exceeding a fold-change ≥1.2 [63], another study reported partially lower mean fold-changes [66]. Further, dysregulation of some miRNAs can only be detected in late stages of disease [30]. Future studies comparing healthy individuals with chronically stressed populations are needed to clarify this distinction.

In addition to identifying individual miRNAs and their response to psychosocial stress, it may be promising to investigate miRNA networks (groups of miRNAs) [22] that may provide greater disease specificity. Most miRNAs are not restricted to a single disease but are detected across various pathologies. However, validation and independent reproducibility of such miRNA signatures remain limited [30].

### 5.2 Participant characteristics

All studies included in this review were conducted in Western countries who are not representative of the global population as a whole and therefore limit the representativeness of all results [90]. One study did not identify stress-related miRNA expression changes, but instead differences in a miRNA host gene between African American women and White women reporting high discrimination [65], emphasizing the importance of diverse samples. Further, most of the studies included in this review examined healthy participants [56,[60], [61], [62], [63],^66^,^67^]. Consequently, it remains unclear whether the observed changes in miRNAs in response to psychosocial stress represent components of an adaptive biological response or pathological processes, potentially contributing to stress-associated diseases. We also reported data on participants with e.g., treatment-resistant major depressive disorder [57] or stress-related disorders [64]. As we did not perform subgroup or sensitivity analyses regarding participants’ health status or medication intake, this heterogeneity limits the generalizability of our synthesis. As miRNA expression can be substantially influenced by diseases e.g., Refs. [32,38] and medication use e.g., Refs. [91,92], it is important to systematically characterize and investigate this in future studies.

Sample sizes were not always, but often small e.g., Refs. [56,63] and may have been insufficient, as only one study reported a power analysis [67]. Although participants’ ages ranged from 18 to 84 years, the average participant was middle-aged. Since some miRNAs correlate with age, the age distribution should be considered in future studies [93]. Further, psychophysiological responses to acute stress (e.g., TSST) may differ depending on age group [94]. Only three studies had a balanced gender distribution between male and female participants [56,65,67], while no non-binary participants were included. Furthermore, only one study accounted for menstrual cycle in women [67]. This should be considered in future studies, as there is evidence that the menstrual cycle influences miRNA expression [95] and that sex-differences in miRNA expression exist in diseases such as metabolic syndrome [96]. While one study excluded women taking oral contraceptives, as well as those who were peri- or post-menopausal or had undergone hysterectomies [59], two studies reported that none of their female participants were taking oral/hormonal contraceptives [56,67]. Given this, although 52.5% of the total sample included here were women, the full spectrum of female participants is not represented.

However, miRNA expression appears to be influenced not only by age and sex, but also by various lifestyle factors such as exercise and diet [97] or smoking [98]. This is consistent with several correlating factors reported in the studies included in this review (see Chapter 4.3.1). Nevertheless, not all studies reported factors such as participant's smoking status, diet, or exercise level. To control potential confounding factors, a standardized assessment of the characteristics and lifestyle habits of participants is essential in miRNA research. It should be further considered that some studies included here did not employ a control group e.g., Ref. [60] which may limit the internal validity.

### 5.3 Psychosocial stress assessment methods

Within this review, studies using various methods to induce and assess psychosocial stress, including laboratory-induced stressors (e.g., TSST) or naturalistic stressors (e.g., exam) as well as various retrospective questionnaires for self-assessment of stress and various biological stress markers (e.g., cortisol or heart rate) were included. This illustrates the range of existing psychosocial stressors, conceptualizations, and assessments.

An experimental, laboratory-induced stressor such as the TSST is a well-validated, standardized stressor for inducing acute psychosocial stress [13,14]. In comparison, a naturalistic stressor such as an examination is automatically closer to the real world but not as standardized. It is uncertain whether the results of studies using laboratory-induced or naturalistic stressors are directly comparable [2] as reactivity might differ [99]. Further, retrospective self-reporting is widely used in stress research and was applied in various ways in the studies included in this review.

Here too, comparisons between studies should be made with caution, as the definition of psychosocial stress differs. For instance, the POMS-2 questionnaire [78] that was used in one of the studies to assess psychological stress, measures the mood states of participants [60], while other studies assessed stressful life events in general [57] or specifically one factor like experiences of discrimination [65]. Questionnaires can cover a broad range of events; therefore, a closer analysis of specific single life events e.g., as reported in [52] may facilitate a greater understanding of associations between miRNAs and psychosocial stress. In addition to subjective data, which may be biased, several of the included studies assessed objective biological stress markers, such as ACTH and salivary cortisol as indicators of HPA axis activity, and heart rate as a marker of the SAM function. Interestingly, not all markers showed the same effect. For instance, no association was found between miR-29c expression and cortisol responsiveness [66], while in another study, only induction of ACTH but not of cortisol levels or cardiovascular measures was significantly correlated with miR-21 induction 1 hour post stressor [59]. Further, one study reported no significant correlation between miRNA levels and state anxiety scores [56], while one study reported significant correlations between miR-16-5p and miR-21-5p with subjectively perceived stress and correlations between alpha-amylase levels and expression of miR-21-5p and miR-26b-5p [67], highlighting the importance of stress measure selection and the necessity for caution when comparing analyses.

Not only did the methods of stress assessment and induction vary widely among the included studies, but timing and duration of psychosocial stress measurements also differed, with some studies focusing on acute stress e.g., Ref. [63] and others on chronic stress e.g., Ref. [58]. However, measurements obtained over an extended period may be influenced by additional factors, which could affect the observed outcome. While an adequate response to acute stress is beneficial, chronic stress in particular can pose a health risk, further leading to immune dysregulation [100]. Further, it is important to highlight, that we did not include ACE and PTSD as psychosocial stressors in this review (for further information see Chapter 3.1), which can limit the generalizability of our findings to adult populations and certain stressors only. However, additional research in this may contribute to a better understanding of the role of miRNAs in the development of stress associated disease. ACEs are associated with greater frequency and severity of daily stressors in adulthood [101] and preliminary findings indicate associations between ACE exposure and salivary miRNA expression [102] as well as miRNA expression in breastmilk [103].

In conclusion, our review highlights the wide variability in psychosocial stress measurements in the field. To improve comparability across studies examining stress-related miRNA changes, it is essential to adopt validated and standardized stress paradigms that comprehensively capture the broad spectrum of psychosocial stress.

### 5.4 miRNA isolation and storage

Altered stress-associated miRNA expression has been investigated in various human body fluids such as blood or saliva (see Table 5). This variety is expected, since miRNAs can also be detected in other body fluids such as tears, urine, and cerebrospinal fluid. Interestingly, they have distinct compositions depending on the type of fluid [40] and considering the different body fluids assessed in the studies presented here, differences should be interpreted with caution. For instance, research from the forensic field showed that miRNA in blood is more resistant to environmental influences compared to miRNA in saliva or semen [104].

Further, miRNA concentrations can vary within the same type of fluid. In serum and saliva, most miRNAs can be identified within the exosome fraction and do not circulate freely. Hence, exosome isolation should be considered, particularly when high sensitivity is required [105]. Although only two studies assessed miRNAs in saliva exosomes [62,67], saliva collection holds great promise for stress research. As a non-invasive procedure, it avoids additional stress associated with blood collection via needle puncture, thus representing a more ethically acceptable approach for biomarker assessment [36]. However, a standardized protocol for the collection and storage of salivary miRNA has yet to be established. Further, the choice of extraction kits and saliva collectors should be tailored to the specific aims of the study [106]. External factors, such as circadian rhythms, can also influence miRNA patterns, making consistent timing of miRNA collection essential [107]. In this review, eight studies reported the timing of miRNA collection [[56], [57], [58], [59], [60],^62^,^63^,^67^]. Further, consistency of storage (e.g., duration or temperature) across studies could improve comparability [107]. Most studies included here did not report the storage duration of samples prior to miRNA isolation or analysis [57,[59], [60], [61], [62], [63], [64], [65], [66], [67]]. Nearly all studies stored miRNA samples at –80°C [[56], [57], [58], [59], [60],^62^,^63^,[65], [66], [67]] or at –20°C [64]. Although miRNAs can remain stable for up to two years under these conditions, prolonged storage may result in a decline of miRNA amount, potentially leading to inaccurate results [107].

### 5.5 miRNA associations with metabolism

Another aim of this review was to examine, on an exploratory basis, the potential associations between stress-related miRNAs and metabolic processes to improve the understanding of underlying cellular mechanisms in the development of stress-related diseases.

Our exploratory search identified links between metabolic diseases (in humans and animals) and over 75% of the miRNAs previously found to be altered in response to psychosocial stress (see Supplementary Table S1). This is not unexpected, given that numerous miRNAs are associated with regulating several metabolic functions, such as cholesterol and lipid homeostasis, insulin signaling, and glucose metabolisms [22]. In line with this, several miRNAs have been proposed as potential disease biomarkers. For instance, miR-21 has been suggested as a tissue-specific biomarker of T2DM, whereas miR-26b is considered as a potential circulating biomarker of T2DM [108]. Notably, miR-21 may even serve as a general biomarker, as it is implicated in both cancer and non-cancerous diseases (involving T2DM or nonalcoholic fatty liver disease) [30]. However, this diversified role can interfere with the development of miRNA therapeutics. For instance, modulating a single miRNA to treat a condition may cause the onset of another disease, as illustrated for miR-144 [109], which has been reported as differentially expressed in response to stress here [56,62]. Although substantial research efforts aim to develop miRNA therapeutic approaches for metabolic diseases such as T2DM, findings remain in early stages. Dysregulated miRNAs could either be restored using miRNA mimics imitating miRNA function or suppressed using miRNA inhibitors. Effective tissue-specific delivery of miRNA or miRNAs with ubiquitous expression patterns throughout all tissues should be addressed in future studies [109]. It is important to highlight that our exploratory investigation for potential associations between stress-related miRNAs and metabolic disorders is preliminary and non-systematic in nature and does not indicate clinical biomarker readiness or provide clinical recommendations. Our search may generate new hypotheses, which can be investigated in future studies.

### 5.6 Strengths and limitations

This review provides a systematic overview of stress-associated miRNA expression changes in humans. 12 studies were included, encompassing a variety of psychosocial stress measurements in different body fluids across different time periods. To minimize bias, two authors independently screened all articles for eligibility, followed by a joint evaluation of methodological quality of all included studies with two different tools. However, it is important to acknowledge that reviews based exclusively on published articles may be susceptible to publication bias, potentially leading to an overestimation of effects [110]. Moreover, it should be noted that our quality assessment yielded mixed findings. Differences in sample characteristics, time points, and stress assessment methods as well as differences in miRNA collection constrain the generalizability and comparability of findings.

### 5.7 Conclusion and future implications

Acute and chronic psychosocial stress can induce changes in miRNA expression in humans. Future research should aim to determine whether these miRNA changes are part of a normal, adaptive biological response to psychosocial stress or whether some changes may be pathological. This can be achieved by comparing non-stressed participants with chronically stressed participants, both with and without stress-related disorders. Such investigations may enable the identification of specific miRNA biomarkers for early diagnosis, prevention, or treatment of stress-related conditions. Particularly promising is the non-invasive collection of miRNAs from saliva for stress research.

Due to the varied methods of stress measurements as well as the use of different body fluids for miRNA collection, and varying observation periods, caution is advised when comparing study results. Standardizing stress assessment methods, miRNA extraction, handling, and laboratory analyses could improve comparability across studies.

Furthermore, most miRNAs associated with psychosocial stress were reported in only a single study and lack replication, which limits the reliability and generalizability of the results. In line with open science principles, additional validation across larger, independent cohorts is necessary to confirm our findings, enhance their overall credibility and to establish their clinical relevance. To better understand the mechanisms underlying stress-related disorders in humans, more systematic research on stress-associated miRNAs in metabolic diseases is needed, as the present review considered only a limited number of miRNA associations and metabolic disorders. Also, addressing the genes associated with differential miRNA expression is crucial for a better understanding of miRNA biomarkers.

## Declaration of generative AI and AI-assisted technologies in the manuscript preparation process

The first author used DeepL and ChatGPT to refine the language of the manuscript. All content was subsequently reviewed and edited by the author(s), who take full responsibility for the final version.

## Funding source

The publication was funded by the Open Access budget of the MSH Medical School Hamburg as part of Project DEAL.

## CRediT authorship contribution statement

**Nele Reinsberg:** Conceptualization, Investigation, Methodology, Validation, Visualization, Writing – original draft, Writing – review & editing. **Ankush Borlepawar:** Conceptualization, Investigation, Validation, Writing – original draft, Writing – review & editing. **Parvana Hajieva:** Conceptualization, Methodology, Supervision, Writing – review & editing. **Anett Müller-Alcazar:** Conceptualization, Methodology, Supervision, Writing – original draft, Writing – review & editing.

## Declaration of competing interest

The authors declare the following financial interests/personal relationships which may be considered as potential competing interests: One of the included studies (Jurkiewicz et al., 2021) 63 was co-authored by AMA. To minimize potential bias, study and data extraction as well as quality assessment for this study were not performed by AMA. If there are other authors, they declare that they have no known competing financial interests or personal relationships that could have appeared to influence the work reported in this paper.

## Appendix A Supplementary Data

- Supplementary Table S1 miRNAs associated with metabolic diseases according to HMDD v.4.0
- Supplementary Table S2. Target Genes of miRNAs associated with psychosocial stress in multiple studies
