# Supplementary material for: Psychosocial stress-associated alterations in microRNAs and their relevance for metabolic diseases: A systematic review
Source: Compr Psychoneuroendocrinol. 2026 Jul 17;27:100364. doi: 10.1016/j.cpnec.2026.100364 (PMC13452199; doi:10.1016/j.cpnec.2026.100364)
Supplement: Supplementary file 1 [file mmc1.docx]

**Supplementary Table S1. miRNAs associated with metabolic diseases according to HMDD v.4.0**

Supplementary Table 1 lists the miRNAs identified in our review as being differentially expressed in response to psychosocial stress in humans. A database search was conducted for these miRNAs in HMDD v4.0 [1] (<https://www.cuilab.cn/hmdd>) between July 2025 and April 2026. Selected exploratory examples of associations with metabolic diseases in humans and/or animals are provided.

**Supplementary Table S1.** miRNAs associated with metabolic diseases according to HMDD v.4.0 [1]

| **miR** | **Metabolic associations reported**  **in HMDD v4.0 [1]** | **Examples of associations with metabolic diseases (exploratory) according to HMDD v4.0 [1]** | **Human Data** | **Animal Data** |
| --- | --- | --- | --- | --- |
| hsa-let-7a-5p | YES | Prediabetes/Diabetes Mellitus Type 2 [2] | X |  |
| hsa-let-7b | YES | Diabetes Mellitus Type 2 (5p strand) [3] | X |  |
| hsa-let-7g-5p | YES | Osteoporosis [4] | X | X |
| hsa-miR-9-5p | YES | Metabolic syndrome [5] | X |  |
| **hsa-miR-10a-5p** | YES | Prediabetes/Diabetes Mellitus Type 2 [2]; Glucose Intolerance/Insulin Resistance [6] | X | X |
| hsa-miR-15a-5p | YES | Insulin Resistance/Overweight [7] | X |  |
| **hsa-miR-16** | YES | Diabetic Kidney Disease (5p strand) [8]  Diabetes Mellitus Type 1 (5p strand) [9] | X | X |
| hsa-miR-19b-3p | YES | Diabetic Nephropathies [10] | X | X |
| **hsa-miR-20b** | YES | Gestational Diabetes Mellitus (5p strand) [11]; Diabetic Retinopathy (5p strand) [12] | X |  |
| **hsa-miR-21** | YES | Metabolic Syndrome (5p strand) [13]; Gestational Diabetes Mellitus (5p strand) [14] | XX | X |
| **hsa-miR-26b** | YES | Diabates Mellitus Type 1 (5p strand) [15]; Diabetes Mellitus Type 2 (5p strand) [16] | XX |  |
| hsa-miR-29a | YES | Gestational Diabetes (3p strand) [17] | X |  |
| hsa-mir-29c | YES | Pregestational and Gestational Obesity (3p strand) [18] | X |  |
| hsa-miR-34a-3p | YES | Obesity (strand not specified) [19] | X |  |
| hsa-miR-92a-2-3p | for hsa-miR-92a-2-5p only | / |  |  |
| hsa-miR-126 | YES | Prediabetes (3p strand) [20] | X |  |
| hsa-miR-129-5p | YES | Obesity [21] | X | X |
| hsa-miR-135-3p | YES | Diabetes Mellitus Type 2 (strand not specified) [22] |  | X |
| hsa-miR-137-3p | YES | Nonalcoholic fatty liver disease [23] |  | X |
| **hsa-miR-144-3p/5p** | YES | Gestational Diabetes Mellitus (strand not specified) [24]  Diabetes Mellitus Type 2 (strand not specified) [25] | XX |  |
| hsa-miR-146a-5p | YES | Diabetes Mellitus Type 1 [26] | X |  |
| hsa-mir-148a-3p | YES | Gestational Diabetes Mellitus [27] | X |  |
| hsa-miR-187-3p | YES | Hepatic Steatosis [28] | X |  |
| hsa-miR-210-3p | YES | Gestational Diabetes Mellitus [29] | X |  |
| hsa-miR-301b | YES | Diabetic Hearts (3p strand) [30] |  | X |
| hsa-miR-375-3p | YES | Non-alcoholic Fatty Liver Disease [31] | X | X |
| hsa-miR-518a-2 | NO | / |  |  |
| hsa-miR-614 | NO | / |  |  |
| hsa-miR-624-3p | for hsa-miR-624-5p only |  |  |  |
| hsa-miR-937-3p | YES | Metabolic Disease (strand not specified) [32] | X |  |
| hsa-miR-1185-5p | NO | / |  |  |
| hsa-miR-3178 | NO | / |  |  |
| hsa-miR-3191-3p | NO | / |  |  |

Note. Abbreviations: HMDD v4.0: Human MicroRNA Disease Database version 4.0; hsa: homo sapiens; miR: microRNA.

bolt letters: miRNAs were identified as differentially expressed in more than one study. *MIR155HG* was not included here, as it is classified as a host gene.

**References**

[1] C. Cui, B. Zhong, R. Fan, Q. Cui, HMDD v4.0: a database for experimentally supported human microRNA-disease associations, Nucleic Acids Res. 52 (2024) D1327-d1332. https://doi.org/10.1093/nar/gkad717.

[2] J. Strycharz, A. Wróblewski, A. Zieleniak, E. Świderska, T. Matyjas, M. Rucińska, L. Pomorski, P. Czarny, J. Szemraj, J. Drzewoski, A. Śliwińska, 2021. Visceral Adipose Tissue of Prediabetic and Diabetic Females Shares a Set of Similarly Upregulated microRNAs Functionally Annotated to Inflammation, Oxidative Stress and Insulin Signaling. Antioxidants. 10, 101. https://doi.org/10.3390/antiox10010101.

[3] I. Kokkinopoulou, E. Maratou, P. Mitrou, E. Boutati, D.C. Sideris, E.G. Fragoulis, M.I. Christodoulou, Decreased expression of microRNAs targeting type-2 diabetes susceptibility genes in peripheral blood of patients and predisposed individuals, Endocrine. 66 (2019) 226-239. https://doi.org/10.1007/s12020-019-02062-0.

[4] R. Chen, X. Liao, F. Chen, B. Wang, J. Huang, G. Jian, Z. Huang, G. Yin, H. Liu, D. Jin, Circulating microRNAs, miR-10b-5p, miR-328-3p, miR-100 and let-7, are associated with osteoblast differentiation in osteoporosis, Int J Clin Exp Pathol. 11 (2018) 1383-1390.

[5] S. D'Amore, J. Härdfeldt, M. Cariello, G. Graziano, M. Copetti, G. Di Tullio, M. Piglionica, N. Scialpi, C. Sabbà, G. Palasciano, M. Vacca, A. Moschetta, Identification of miR-9-5p as direct regulator of ABCA1 and HDL-driven reverse cholesterol transport in circulating CD14+ cells of patients with metabolic syndrome, Cardiovasc Res. 114 (2018) 1154-1164. https://doi.org/10.1093/cvr/cvy077.

[6] Y. Guo, X. Zhu, S. Zeng, M. He, X. Xing, C. Wang, 2020. miRNA-10a-5p Alleviates Insulin Resistance and Maintains Diurnal Patterns of Triglycerides and Gut Microbiota in High-Fat Diet-Fed Mice. Mediators Inflamm. 2020, 8192187. https://doi.org/10.1155/2020/8192187.

[7] F. Ramzan, R.F. D'Souza, B.R. Durainayagam, A.M. Milan, N.C. Roy, M.C. Kruger, C.J. Henry, C.J. Mitchell, D. Cameron-Smith, 2020. Inflexibility of the plasma miRNA response following a high-carbohydrate meal in overweight insulin-resistant women. Genes Nutr. 15, 2. https://doi.org/10.1186/s12263-020-0660-8.

[8] T.S. Assmann, M. Recamonde-Mendoza, A.R. Costa, M. Puñales, B. Tschiedel, L.H. Canani, A.C. Bauer, D. Crispim, Circulating miRNAs in diabetic kidney disease: case-control study and in silico analyses, Acta Diabetol. 56 (2019) 55-65. https://doi.org/10.1007/s00592-018-1216-x.

[9] X. Gao, S. Zhao, miRNA-16-5p inhibits the apoptosis of high glucose-induced pancreatic β cells via targeting of CXCL10: potential biomarkers in type 1 diabetes mellitus, Endokrynol Pol. 71 (2020) 404-410. https://doi.org/10.5603/EP.a2020.0046.

[10] L.L. Lv, Y. Feng, M. Wu, B. Wang, Z.L. Li, X. Zhong, W.J. Wu, J. Chen, H.F. Ni, T.T. Tang, R.N. Tang, H.Y. Lan, B.C. Liu, Exosomal miRNA-19b-3p of tubular epithelial cells promotes M1 macrophage activation in kidney injury, Cell Death Differ. 27 (2020) 210-226. https://doi.org/10.1038/s41418-019-0349-y.

[11] I. Hromadnikova, K. Kotlabova, L. Krofta, 2022. Cardiovascular Disease-Associated MicroRNAs as Novel Biomarkers of First-Trimester Screening for Gestational Diabetes Mellitus in the Absence of Other Pregnancy-Related Complications. International Journal of Molecular Sciences. 23, 10635. https://doi.org/10.3390/ijms231810635.

[12] M.C. Trotta, C. Gesualdo, C.B.M. Platania, D. De Robertis, M. Giordano, F. Simonelli, M. D'Amico, F. Drago, C. Bucolo, S. Rossi, 2021. Circulating miRNAs in diabetic retinopathy patients: Prognostic markers or pharmacological targets? Biochemical Pharmacology. 186, 114473. https://doi.org/10.1016/j.bcp.2021.114473.

[13] A.S. Doghish, A.M. Elsisi, A.I. Amin, A.I. Abulsoud, Circulating miR-148a-5p and miR-21-5p as Novel Diagnostic Biomarkers in Adult Egyptian Male Patients With Metabolic Syndrome, Can J Diabetes. 45 (2021) 614-618. https://doi.org/10.1016/j.jcjd.2020.12.005.

[14] C.Y. Guan, S. Tian, J.L. Cao, X.Q. Wang, X. Ma, H.F. Xia, Down-Regulated miR-21 in Gestational Diabetes Mellitus Placenta Induces PPAR-α to Inhibit Cell Proliferation and Infiltration, Diabetes Metab Syndr Obes. 13 (2020) 3009-3034. https://doi.org/10.2147/dmso.S253920.

[15] R.S. Ferraz, L.C.B. Santos, R.L. da-Silva-Cruz, C.H. Braga-da-Silva, L. Magalhães, A. Ribeiro-dos-Santos, A. Vidal, T. Vinasco-Sandoval, L. Reis-das-Mercês, C. Sena-dos-Santos, A.L. Pereira, L.S.D.A. Silva, F.T.C.d. Melo, A.C.C.B.d. Souza, V.S.G. Leal, P.B.B.d. Figueiredo, J.F.A. Neto, L.V.d. Moraes, G.N.d. Lemos, N.N.M.d. Queiroz, K.M. Felício, G.C. Cavalcante, Â. Ribeiro-dos-Santos, J.S. Felício, 2022. Global miRNA expression reveals novel nuclear and mitochondrial interactions in Type 1 diabetes mellitus. Frontiers in Endocrinology. Volume 13 - 2022, 1033809. https://doi.org/10.3389/fendo.2022.1033809.

[16] H. Demirsoy İ, D.Y. Ertural, Ş. Balci, Ü. Çınkır, K. Sezer, L. Tamer, N. Aras, Profiles of Circulating MiRNAs Following Metformin Treatment in Patients with Type 2 Diabetes, J Med Biochem. 37 (2018) 499-506. https://doi.org/10.2478/jomb-2018-0009.

[17] A. Martínez-Ibarra, L.D. Martínez-Razo, E.R. Vázquez-Martínez, N. Martínez-Cruz, R. Flores-Ramírez, E. García-Gómez, M. López-López, C. Ortega-González, I. Camacho-Arroyo, M. Cerbón, 2019. Unhealthy Levels of Phthalates and Bisphenol A in Mexican Pregnant Women with Gestational Diabetes and Its Association to Altered Expression of miRNAs Involved with Metabolic Disease. International Journal of Molecular Sciences. 20. https://doi.org/10.3390/ijms20133343.

[18] G. Carreras-Badosa, A. Bonmatí, F.J. Ortega, J.M. Mercader, M. Guindo-Martínez, D. Torrents, A. Prats-Puig, J.M. Martinez-Calcerrada, E. Platero-Gutierrez, F. De Zegher, L. Ibáñez, J.M. Fernandez-Real, A. Lopez-Bermejo, J. Bassols, Altered Circulating miRNA Expression Profile in Pregestational and Gestational Obesity, J Clin Endocrinol Metab. 100 (2015) E1446-1456. https://doi.org/10.1210/jc.2015-2872.

[19] M. Zarkesh, K. Tabaei, M. Akbarzadeh, A. Daneshafrooz, A. Zadeh-Vakili, 2022. Association of miR-34a and miR-143 levels with PPARγ gene expression in adipose tissues of non-diabetic adults. J Physiol Anthropol. 41, 13. https://doi.org/10.1186/s40101-022-00286-0.

[20] T.E. Matsha, A.P. Kengne, S. Hector, D.L. Mbu, Y.Y. Yako, R.T. Erasmus, MicroRNA profiling and their pathways in South African individuals with prediabetes and newly diagnosed type 2 diabetes mellitus, Oncotarget. 9 (2018) 30485-30498. https://doi.org/10.18632/oncotarget.25271.

[21] X. Fu, L. Jin, L. Han, Y. Yuan, Q. Mu, H. Wang, J. Yang, G. Ning, D. Zhou, Z. Zhang, 2019. miR-129-5p Inhibits Adipogenesis through Autophagy and May Be a Potential Biomarker for Obesity. Int J Endocrinol. 2019, 5069578. https://doi.org/10.1155/2019/5069578.

[22] M. Honardoost, F. Keramati, E. Arefian, S. Mohammadi Yeganeh, M. Soleimani, Network of three specific microRNAs influence type 2 diabetes through inducing insulin resistance in muscle cell lines, J Cell Biochem. 120 (2019) 1532-1538. https://doi.org/10.1002/jcb.27381.

[23] Y. Yu, C. He, S. Tan, M. Huang, Y. Guo, M. Li, Q. Zhang, 2021. MicroRNA-137-3p Improves Nonalcoholic Fatty Liver Disease through Activating AMPKα. Anal Cell Pathol (Amst). 2021, 4853355. https://doi.org/10.1155/2021/4853355.

[24] L. Zhang, T. Zhang, D. Sun, G. Cheng, H. Ren, H. Hong, L. Chen, X. Jiao, Y. Du, Y. Zou, L. Wang, Diagnostic value of dysregulated microribonucleic acids in the placenta and circulating exosomes in gestational diabetes mellitus, J Diabetes Investig. 12 (2021) 1490-1500. https://doi.org/10.1111/jdi.13493.

[25] E. Flowers, B.E. Aouizerat, A.M. Kanaya, J.C. Florez, X. Gong, L. Zhang, MicroRNAs Associated With Incident Diabetes in the Diabetes Prevention Program, J Clin Endocrinol Metab. 108 (2023) e306-e312. https://doi.org/10.1210/clinem/dgac714.

[26] C. Frørup, A.H. Mirza, R. Yarani, L.B. Nielsen, E.R. Mathiesen, P. Damm, J. Svare, C. Engelbrekt, J. Størling, J. Johannesen, H.B. Mortensen, F. Pociot, S. Kaur, 2021. Plasma Exosome-Enriched Extracellular Vesicles From Lactating Mothers With Type 1 Diabetes Contain Aberrant Levels of miRNAs During the Postpartum Period. Frontiers in Immunology. Volume 12 - 2021, 744509. https://doi.org/10.3389/fimmu.2021.744509.

[27] Z. Ye, S. Wang, X. Huang, P. Chen, L. Deng, S. Li, S. Lin, Z. Wang, B. Liu, Plasma Exosomal miRNAs Associated With Metabolism as Early Predictor of Gestational Diabetes Mellitus, Diabetes. 71 (2022) 2272-2283. https://doi.org/10.2337/db21-0909.

[28] X. Zhang, M.M.J. Mens, Y.J. Abozaid, D. Bos, S. Darwish Murad, R.J. de Knegt, M.A. Ikram, Q. Pan, M. Ghanbari, Circulatory microRNAs as potential biomarkers for fatty liver disease: the Rotterdam study, Aliment Pharmacol Ther. 53 (2021) 432-442. https://doi.org/10.1111/apt.16177.

[29] V. Gillet, A. Ouellet, Y. Stepanov, R.S. Rodosthenous, E.K. Croft, K. Brennan, N. Abdelouahab, A. Baccarelli, L. Takser, miRNA Profiles in Extracellular Vesicles From Serum Early in Pregnancies Complicated by Gestational Diabetes Mellitus, J Clin Endocrinol Metab. 104 (2019) 5157-5169. https://doi.org/10.1210/jc.2018-02693.

[30] Y. Tatekoshi, M. Tanno, H. Kouzu, K. Abe, T. Miki, A. Kuno, T. Yano, S. Ishikawa, W. Ohwada, T. Sato, T. Niinuma, H. Suzuki, T. Miura, Translational regulation by miR-301b upregulates AMP deaminase in diabetic hearts, J Mol Cell Cardiol. 119 (2018) 138-146. https://doi.org/10.1016/j.yjmcc.2018.05.003.

[31] X. Chen, W. Xue, J. Zhang, J. Peng, W. Huang, 2023. Ginsenoside Rg1 attenuates the NASH phenotype by regulating the miR-375-3p/ATG2B/PTEN-AKT axis to mediate autophagy and pyroptosis. Lipids Health Dis. 22, 22. https://doi.org/10.1186/s12944-023-01787-2.

[32] C. Yang, J. Guo, Y. Lin, G. Hu, J. Wie, X. Zhang, J. Sun, J. Li, Peripheral Blood miR-937 May Serve as a Biomarker for Metabolic Disorders by Targeting AMPKα, Clin Lab. 65 (2019). https://doi.org/10.7754/Clin.Lab.2018.181113.
