# Supplementary material for: Psychosocial stress-associated alterations in microRNAs and their relevance for metabolic diseases: A systematic review
Source: Compr Psychoneuroendocrinol. 2026 Jul 17;27:100364. doi: 10.1016/j.cpnec.2026.100364 (PMC13452199; doi:10.1016/j.cpnec.2026.100364)
Supplement: Supplementary file 2 [file mmc2.docx]

**Supplementary Table S2. Target Genes of miRNAs associated with psychosocial stress in multiple studies.**

Supplementary Table S2. Target Genes of hsa-miR-10a-5p, hsa-miR-16-5p, hsa-miR-20b-5p, hsa-miR-21-5p, hsa-miR-26b-5p, hsa-miR-144-3p and hsa-miR-144-5p according to a miRTarBase (Version 2025) search conducted in March and April 2026 [1].

**Supplementary Table S2. Target Genes of miRNAs associated with psychosocial stress in multiple studies.**

| **miR** | **Target Genes according to mirTarBase Version 2025 [1]; strong evidence validation methods only (Reporter Assay, Western blot, qPCR)** | |
| --- | --- | --- |
| **hsa-miR-10a-5p** | ARNTL |  |
|  | BDNF |  |
|  | EPHA4 |  |
|  | FASN |  |
|  | HOXA1 |  |
|  | SERPINE1 |  |
|  | PIK3CG |  |
|  | MAP2K6 |  |
|  | RORA |  |
|  | SDC1 |  |
|  | MAP3K7 |  |
|  | USF2 |  |
|  | SKA1 |  |
|  |  |  |
| **hsa-miR-16-5p** | ADRB2 |  |
|  | APP |  |
|  | ARL2 |  |
|  | CCND1 |  |
|  | BCL2 |  |
|  | BDNF |  |
|  | CCND3 |  |
|  | CCNE1 |  |
|  | CDK6 |  |
|  | CDKN1A |  |
|  | CHUK |  |
|  | ACSL1 |  |
|  | FGF2 |  |
|  | FGFR1 |  |
|  | HDGF |  |
|  | HMGA1 |  |
|  | IGF1R |  |
|  | IRAK2 |  |
|  | KDR |  |
|  | CAPRIN1 |  |
|  | NOTCH2 |  |
|  | TLN1 |  |
|  | VEGFA |  |
|  | ZYX |  |
|  | HMGA2 |  |
|  | AXIN2 |  |
|  | RECK |  |
|  | PPM1D |  |
|  | CDS2 |  |
|  | MAP7 |  |
|  | YAP1 |  |
|  | PRDM4 |  |
|  | NCSTN |  |
|  | BACE1 |  |
|  | SOX6 |  |
|  | Ccnd1 |  |
|  |  |  |
| **hsa-miR-20b-5p** | CDKN1A |  |
|  | E2F1 |  |
|  | ESR1 |  |
|  | PTEN |  |
|  | RB1 |  |
|  | VEGFA |  |
|  | WNT9B |  |
|  | BAMBI |  |
|  | TP53INP1 |  |

**Supplementary Table 2.** *(continued)*

| **miR** | **Target Genes according to mirTarBase Version 2025 [1]; strong evidence validation methods only (Reporter Assay, Western blot, qPCR)** |
| --- | --- |
| **hsa-miR-21-5p** | APAF1 |
|  | FASLG |
|  | RHOB |
|  | BCL2 |
|  | BMPR2 |
|  | CDK6 |
|  | CCR7 |
|  | DAXX |
|  | E2F1 |
|  | EGFR |
|  | EIF4A2 |
|  | FOXO3 |
|  | MSH6 |
|  | HPGD |
|  | IL12A |
|  | IRAK1 |
|  | SMAD7 |
|  | MMP9 |
|  | MSH2 |
|  | MTAP |
|  | MYD88 |
|  | NFIB |
|  | PFKFB2 |
|  | SERPINB5 |
|  | SERPINI1 |
|  | SMARCA4 |
|  | SOX5 |
|  | STRN |
|  | TGFBR2 |
|  | TPM1 |
|  | VHL |
|  | CDK2AP1 |
|  | ANP32A |
|  | GDF5 |
|  | RECK |
|  | TP63 |
|  | LRRFIP1 |
|  | DNM1L |
|  | RASGRP1 |
|  | PIAS3 |
|  | DUSP10 |
|  | ISCU |
|  | DDAH1 |
|  | CDIP1 |
|  | SOX6 |
|  | SOX17 |
|  | WWC2 |
|  | SLC16A10 |
|  | ANKRD46 |
|  | CASC2 |
|  |  |
| **hsa-miR-26b-5p** | CD38 |
|  | COL1A2 |
|  | ATF2 |
|  | CTGF |
|  | EPHA2 |
|  | EZH2 |
|  | GATA4 |
|  | KPNA2 |
|  | COX2 |
|  | PRKCD |
|  | PTGS2 |
|  | RB1 |
|  | NR2C2 |
|  | USP9X |
|  | NAMPT |
|  | TAB1 |
|  |  |

**Supplementary Table 2.** *(continued)*

| **miR** | **Target Genes according to mirTarBase [1] strong evidence validation methods**  **(Reporter Assay, Western blot, qPCR)** |
| --- | --- |
| **hsa-miR-144-3p** | ADAM10 |
|  | BCL6 |
|  | MAP3K8 |
|  | EZH2 |
|  | MET |
|  | NOTCH1 |
|  | PTEN |
|  | PTGS2 |
|  | ZEB1 |
|  | TGFB1 |
|  | TTN |
|  | ZFX |
|  | ZEB2 |
|  | ADAMTS5 |
|  | FBXW7 |
|  |  |
| **hsa-miR-144-5p** | SMAD1 |

Note. Only target genes with strong evidence validation methods considered (Reporter Assay, Western blot and qPCR) in mirTarBase search.

Abbreviations: hsa: homo sapiens; miR: microRNA; qPCR: quantitative polymerase chain reaction

**References**

[1] S. Cui, S. Yu, H.Y. Huang, Y.C. Lin, Y. Huang, B. Zhang, J. Xiao, H. Zuo, J. Wang, Z. Li, G. Li, J. Ma, B. Chen, H. Zhang, J. Fu, L. Wang, H.D. Huang, miRTarBase 2025: updates to the collection of experimentally validated microRNA-target interactions, Nucleic Acids Res. 53 (2025) D147-d156. https://doi.org/10.1093/nar/gkae1072.
